# Thyroid hormone profile is related to prognosis in acute decompensation of cirrhosis

**DOI:** 10.20945/2359-4292-2023-0249

**Published:** 2024-08-13

**Authors:** Giovana De Nardin, Bruno da Silveira Colombo, Marcelo Fernando Ronsoni, Pedro Eduardo Soares e Silva, Leonardo Fayad, Letícia Muraro Wildner, Maria Luiza Bazzo, Esther Buzaglo Dantas-Correa, Janaína Luz Narciso-Schiavon, Leonardo de Lucca Schiavon

**Affiliations:** 1 Divisão de Gastroenterologia Universidade Federal de Santa Catarina Florianópolis SC Brasil Divisão de Gastroenterologia, Universidade Federal de Santa Catarina, Florianópolis, SC, Brasil; 2 Departamento de Análises Clínicas Universidade Federal de Santa Catarina Florianópolis SC Brasil Departamento de Análises Clínicas, Universidade Federal de Santa Catarina, Florianópolis, SC, Brasil

**Keywords:** Liver cirrhosis, acute decompensation, thyroid hormones

## Abstract

**Objective:**

To investigate the prognostic significance of thyroid hormone profile in patients hospitalized for decompensated cirrhosis.

**Subjects and methods:**

Prospective cohort study that included 119 subjects. All subjects were evaluated at admission and followed for 90 days. TSH, fT3, fT4 were measured within 24 hours of hospitalization.

**Results:**

Higher fT4 and lower fT3 levels were observed among Child-Pugh C patients as compared to Child-Pugh A and B, and in those with acute-on-chronic liver failure (ACLF). Lower fT3/fT4 ratio was observed in those with ascites, infections, ACLF, and in Child-Pugh C. Ninety-day mortality was 26.9% and it was independently associated with higher Model for End-stage Liver Disease (MELD) and TSH, and lower fT3/fT4 ratio in multivariate analysis. A new prognostic model including MELD, TSH and fT3/fT4 ratio was devised. The areas under the receiver operating characteristic curves for MELD, fT3/fT4 ratio, TSH (μIU/mL), and the new model for predicting 90-day mortality were 0.847 ± 0.041, 0.841 ± 0.039, 0.658 ± 0.062, and 0.899 ± 0.031, respectively. The 90-day survival was 31.6% in patients with values of the predictive model ≥ -0.77 and 93.5% for values < -0.77 (P < 0.001).

**Conclusions:**

Thyroid hormone profile was strongly associated with worse outcomes in patients with cirrhosis and might represent promising prognostic tools that can be incorporated in clinical practice.

## INTRODUCTION

Cirrhosis is a pathological diagnosis characterized by diffuse fibrosis, severe disruption of the intrahepatic arterial and venous flow, portal hypertension, and, ultimately, liver failure (1). In Europe, liver cirrhosis is responsible for around 170,000 deaths per year, and more than 5500 liver transplants are performed each year (2). The natural history of cirrhosis is usually characterized by a long-standing phase of compensated disease, when the risk of liver-related death is low, followed by a decompensated phase when mortality increases significantly along with the development of complications such as ascites, hepatic encephalopathy, variceal bleeding, and bacterial infection (3). The annual risk of death by liver cirrhosis varies from as low as 1% to as high as 57% depending on the presence of clinically relevant portal hypertension and the number and type of complications observed (3).

The liver plays a central role in the thyroid hormone metabolism, being involved in conjugation, excretion, peripheral deiodination, and the synthesis of thyroxine (T4) binding globulin (4). Therefore, several thyroid abnormalities in patients with chronic liver diseases were described, ranging from morphological changes to alterations in thyroid hormone metabolism and regulation (5,6). The most common thyroid hormone abnormality described in cases of liver cirrhosis has been the low triiodothyronine (T3) pattern, also known as euthyroid sick syndrome (ESS), likely as a result of impaired hepatic uptake and conversion of T4 to T3 as a result of liver failure, but also suppression of hypothalamic thyrotropin-releasing hormone resulting from systemic inflammation (7-9).

ESS is typically characterized by low T3 with normal thyroid stimulating hormone (TSH) levels and increased reverse triiodothyronine levels (9). A decreased free T3 to free T4 ratio (fT3/fT4) is also described in EES and was recently shown to be able to differentiate central hypothyroidism from ESS in children and adolescents (10). Low T3 pattern has been shown to be related to poor outcomes in some earlier series of patients with cirrhosis (11,12), and the fT3/fT4 ratio was recently associated with more severe liver disease and ACLF, although its prognostic significance is unclear (13). As the liver is an important site for T4 to T3 conversion, we hypothesized that combining both hormones in the fT3/fT4 ratio will better reflect the magnitude of thyroid hormone imbalance in liver cirrhosis. We aimed to investigate the prognostic significance of the fT3, fT4, TSH, and fT3/fT4 ratio in patients hospitalized for acute decompensation of cirrhosis.

## SUBJECTS AND METHODS

### Patients

This prospective cohort study is part of a project that aims to follow a cohort of adult patients (≥18 years of age) admitted to the emergency room of a Brazilian tertiary hospital due to AD of liver cirrhosis. Details about the methodology were previously published (14) and are briefly presented below.

All consecutive subjects admitted to the emergency room between January 2011 and November 2013 were evaluated for inclusion. The following exclusion criteria were adopted: hospitalization for elective procedures; admissions not related to complications of liver cirrhosis; hepatocellular carcinoma outside Milan criteria; known thyroid diseases at the time of admission; medications that affect thyroid function (interferon, lithium, and amiodarone); (15) and doubtful diagnosis of liver cirrhosis. The diagnosis of cirrhosis was established either histologically (when available) or by the combination of clinical, imaging, and laboratory findings in patients with evidence of portal hypertension.

The study protocol complies with the ethical principles of the Declaration of Helsinki and was approved by the Ethics Committee on Human Research of the Federal University of Santa Catarina.

### Methods

All patients admitted for AD, as defined by the acute development of hepatic encephalopathy, large ascites, gastrointestinal bleeding, bacterial infection, or any combination of these, were screened. Patients were evaluated within 24 hours of admission by one of the researchers involved in the study, and the following clinical variables were collected: age, gender, race, etiology of cirrhosis, history of previous decompensation, current complications of cirrhosis, and active alcoholism. All subjects underwent laboratory evaluation at admission, and the following tests were performed for this study: total leukocytes, serum sodium, creatinine, international normalized ratio (INR), albumin, C-reactive protein (CRP), and total bilirubin.

Active alcoholism was defined as an average overall consumption of 21 or more drinks per week for men and 14 or more drinks per week for women during the 4 weeks before enrollment (one standard drink is equal to 12 g of absolute alcohol) (16). The patients were followed during their hospital stay. 90-day mortality was verified by phone call, in case of hospital discharge. 90-day mortality rates were considered transplant-free mortality (patients who received a liver transplant were considered lost to follow-up).

Individuals with a suspected infection at the time of hospital admission received a clinical examination to confirm such diagnosis and to establish the primary source of infection. The diagnosis of infection was made according to the criteria of the Center for Disease Control (17). A diagnostic paracentesis was performed for all patients with ascites at admission. Spontaneous bacterial peritonitis (SBP) was diagnosed when the neutrophil count of the ascitic fluid was ≥ 250 neutrophils/mm^3^ in the absence of an intra-abdominal source of infection, regardless of negative culture (18). All patients with SBP received antibiotics plus weight-based intravenous albumin on the first and third day after diagnosis. Hepatic encephalopathy was graded according to the West-Haven criteria (19) and, if present, a precipitant event was actively investigated, lactulose was initiated, and the dosage was adjusted as needed. All subjects with acute variceal bleeding received intravenous octreotide, an antibiotic (either oral quinolone or intravenous ceftriaxone), and underwent urgent therapeutic endoscopy after stabilization. The severity of liver disease was estimated by the Child-Pugh classification system (20) and Model for End-Stage Liver Disease (MELD) (21) calculated based on laboratory tests performed at admission. Acute-on-chronic liver failure (ACLF) was defined as proposed by the EASL-CLIF Consortium (22).

### Thyroid hormone levels

Thyroid hormone levels were measured in samples collected within 24 hours of admission by Chemiluminescent Microparticle Immunoassay (ADVIA Centaur XP^®^ Immunoassay System, Siemens Healthcare, Munich, Germany). The reference range was between 0.4 μIU/mL and 4.00 μIU/mL for TSH; between 1.80 pg/mL to 4.2 pg/mL for fT3; and between 0.89 ng/dL to 1.76 ng/dL for fT4.

### Statistical analysis

The normality of the variable distribution was determined using the Kolmogorov-Smirnov test. Spearman’s coefficient was used for correlations between two continuous variables. Continuous variables were compared using the Student’s *t*-test in the case of a normal distribution or the Mann-Whitney test in the remaining cases. Categorical variables were evaluated by chi-square test or Fisher’s exact test, as appropriate. After checking for multicollinearity among potential independent predictors, multiple logistic regression analysis (forward stepwise regression) was used to investigate the factors independently associated with 90-day mortality. A predictive model was devised including variables with prognostic significance in the regression analysis. Based on the receiver operating characteristics (ROC) curve, the best cutoffs for the variables to predict 90-day mortality were chosen. The survival curve was calculated using the Kaplan-Meier method, and survival differences between groups were compared using the log-rank test. All tests were performed using SPSS software, version 22.0 (SPSS, Chicago, IL, USA). A *p* value of less than 0.05 was considered statistically significant.

## RESULTS

### Characteristics of the sample

Characteristics of the included patients are shown in Table 1. The study included 119 patients hospitalized for acute decompensation of cirrhosis. The mean age was 53.54 ± 12.13 years, 63.9% were Caucasians, and a male predominance was observed (71.4%). Previous history of cirrhosis decompensation was observed in 61.3% of the sample, and 35.3% of the subjects reported active alcoholism during the past month. The mean MELD score was 16.23 ± 6.42 and 38.7% of the subjects were Child-Pugh C.

**Table 1:** Characteristics of included patients

	Patients (n = 119)
Age; years, mean ± SD	53.54 ± 12.13
Caucasians, n (%)	76 (63.9)
Male gender, n (%)	85 (71.4)
Etiology of cirrhosis, n (%)	
Alcohol	43 (36.1)
Hepatitis C	48 (40.3)
Hepatitis B	5 (4.2)
NASH	6 (5.0)
Autoimmune	7 (5.9)
Other	10 (8.4)
Previous decompensation, n (%)	73 (61.3)
Active alcoholism, n (%)	42 (35.3)
Complication at evaluation, n (%)	
Ascites	55 (46.2)
Hepatic encephalopathy	66 (55.5)
Gastrointestinal bleeding	67 (56.3)
Bacterial infection	27 (22.7)
ACLF	28 (23.8)
Laboratory data	
Leucocyte count (x 10^9^), median (IQR)	7.25 (4.84-10.21)
Sodium (meq/L), mean ± SD	135.54 ± 6.16
Creatinine (mg/dl), median (IQR)	1.00 (0.80-1.40)
INR, median (IQR)	1.43 (1.28-1.65)
Albumin (g/dL), mean ± SD	2.35 ± 0.64
CRP (mg/L), median (IQR)	10.10 (3.80-34.70)
Total bilirubin (mg/dL), median (IQR)	1.9 (0.90-3.85)
Child-Pugh Classification, n (%)	
A	15 (12.6)
B	58 (48.7)
C	46 (38.7)
MELD score, mean ± SD	16.23 ± 6.42
Thyroid hormones	
TSH (μIU/mL), median (IQR)	1.26 (0.58-2.81)
fT3 (pg/mL), mean ± SD	1.99 ± 0.79
fT4 (ng/dL), mean ± SD	1.12 ± 0.23
fT3/fT4 ratio, mean ± SD	1.87 ± 0.92

SD: standard deviation; NASH: non-alcoholic steatohepatitis; ACFL: acute-on-chronic liver failure; IQR: interquartile range; INR: international normalized ratio; CRP: C-reactive protein; MELD: Model for End-stage Liver Disease; TSH: thyroid stimulating hormone; fT3: free triiodothyronine; fT4: free thyroxine.

Upon admission, upper gastrointestinal bleeding was observed in 56.3% of cases, ascites in 46.2%, hepatic encephalopathy in 55.5%, bacterial infections in 22.7%, and ACLF in 23.5%.

### The relationship between thyroid hormones and the studied variables

Median TSH and mean fT3 and fT4 levels are shown in Table 1. TSH levels higher than 4.00 μIU/mL or lower than 0.4 μIU/mL were observed in 12% and 16% of the patients, respectively. FT3 was lower than 1.80 pg/mL in 45% of the patients and fT4 was lower than 0.89 ng/dL in 4% of the subjects. No patients exhibited fT3 or fT4 higher than the upper limit of normal.

**Figure 1:**
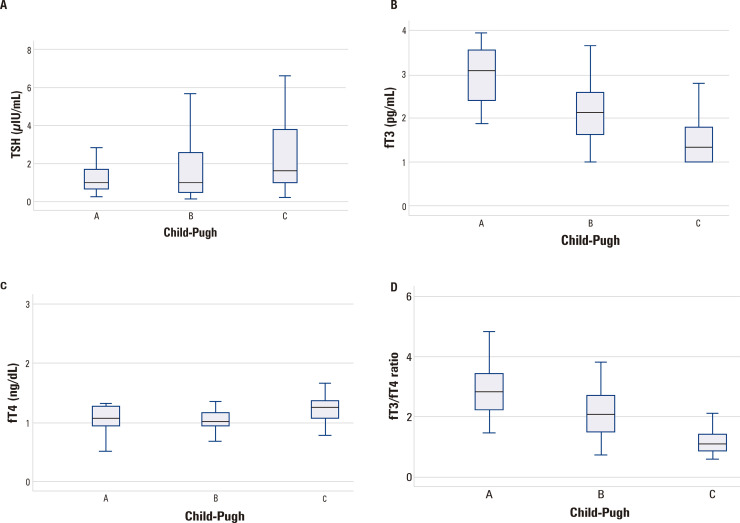
Thyroid hormones profile according to Child-Pugh classification. Significantly higher TSH levels were observed in Child-Pugh C patients as compared to Child-Pugh B (P = 0.033), but not for comparison between Child-Pugh A and C (P = 0.099; Figure 1A). Lower fT3 was observed in Child-Pugh C as compared to Child-Pugh A (P < 0.001) and Child-Pugh B (P < 0.001); also for Child-Pugh B than Child-Pugh A (P = 0.001; Figure 1B). Higher fT4 levels were observed in Child-Pugh C patients as compared to Child-Pugh A (P = 0.008) and Child-Pugh B (P < 0.001), but not for comparison between Child-Pugh A and B (Figure 1C). Significantly lower fT3/fT4 values were observed in Child-Pugh C patients as compared to Child-Pugh A (P < 0.001) and Child-Pugh B (P < 0.001). Child-Pugh B patients also present with lower fT3/fT4 ratio than Child-Pugh A (P = 0.003; Figure 1D).

**Figure 2:**
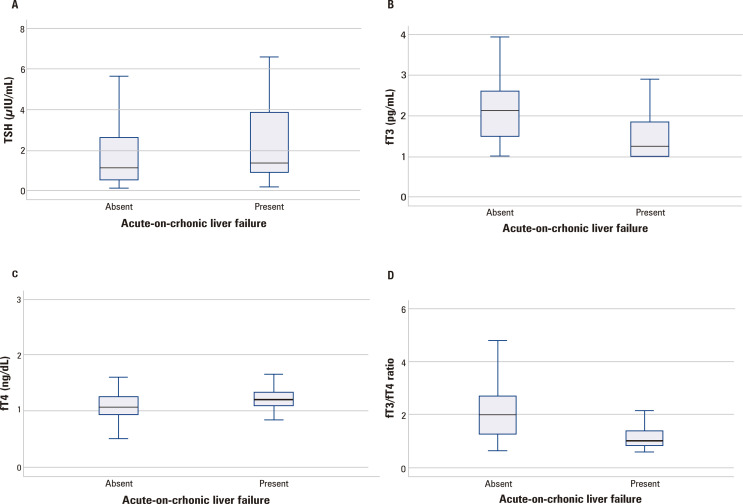
Thyroid hormones profile according to the presence of ACLF at admission. No differences were observed for TSH levels according to ACLF (P = 0.196) (Figure 1D). Lower fT3 (Figure 2B; P < 0.001) and higher fT4 levels (Figure 2C; P = 0.001), as well as lower fT3/fT4 ratio (Figure 2D; P < 0.001) were observed among patients with ACLF.

Correlations between thyroid hormones and numerical variables of interest are shown in Table 2. Thyroid hormone parameters were also evaluated according to Child-Pugh Classification (Figure 1) and regarding the presence of ACLF (Figure 2).

**Table 2:** Spearman’s correlation coefficient between TSH, fT3, fT4, fT3/fT4 ratio and numerical variables

Variable	TSH		fT3		fT4		fT3/fT4	
	r	P	r	P	r	P	r	P
TSH	-	-	-0.27	0.773	0.263	0.004	-0.137	0.144
fT3	-0.027	0.773	-	-	-0.248	0.007	0.908	<0.001
fT4	0.263	0.004	-0.248	0.007	-	-	-0.603	<0.001
fT3/fT4	-0.137	0.144	0.908	<0.001	-0.603	<0.001	-	-
Age	0.175	0.061	0.005	0.954	0.188	0.041	-0.066	0.478
Leukocyte count	- 0.006	0.952	-0.139	0.133	0.061	0.515	-0.130	0.162
Creatinine	0.104	0.267	-0.298	0.001	0.179	0.053	-0.316	<0.001
Total Bilirubin	0.098	0.299	-0.393	<0.001	-0.401	<0.001	-0.483	<0.001
Albumin	-0.117	0.218	0.473	<0.001	-0.202	0.032	0.467	<0.001
INR	0.052	0.581	-0.464	<0.001	0.190	0.040	-0.465	<0.001
Sodium	-0.162	0.083	0.385	<0.001	-0.290	0.001	0.435	<0.001
CRP	0.301	0.001	-0.382	<0.001	0.348	<0.001	-0.434	<0.001
MELD	0.157	0.092	-0.530	<0.001	0.435	<0.001	-0.612	<0.001

INR: international normalized ratio; CRP: C-reactive protein; MELD: Model for End-Stage Liver Disease.

TSH levels were positively correlated with fT4 and CPR. Significantly higher median levels of TSH were observed in Child-Pugh C (1.63 μIU/mL) patients as compared to Child-Pugh B ones (0.99 μIU/mL; P = 0.033), but not for comparison between Child-Pugh A and C (1.63 μIU/mL vs. 1.00 μIU/mL; P = 0.099), (Figure 1A). Lower TSH levels were observed in those with upper gastrointestinal bleeding (0.71 μIU/mL *vs.* 2.54 μIU/mL, P < 0.001). No differences were observed in TSH levels according to ACLF (P = 0.196), (Figure 2A).

FT3 levels were positively correlated with sodium, albumin, and fT3/fT4 ratio. A negative correlation was observed between fT3 levels and fT4, lNR, total bilirubin, CRP, MELD, and creatinine. Significantly lower levels of fT3 were observed in Child-Pugh C patients as compared to Child-Pugh A (1.50 ± 0.59 pg/mL *vs.* 2.95 ± 0.70 pg/mL, P < 0.001) and Child-Pugh B ones (1.50 ± 0.59 pg/mL *vs.* 2.12 ± 0.67 pg/mL; P < 0.001). Child-Pugh B patients also presented lower fT3 than Child-Pugh A ones (2.12 ± 0.67 pg/mL *vs.* 2.95 ± 0.70, P = 0.001; Figure 1B). Lower fT3 levels were also noted in patients with ascites (1.63 ± 0.65 pg/mL *vs.* 2.29 ± 0.78 pg/mL; P < 0.001), hepatic encephalopathy (1.77 ± 0.71 pg/mL *vs.* 2.26 ± 0.81 pg/mL; P = 0.001), ACLF (1.48 ± 0.58 pg/mL *vs.* 2.14 ± 0.79 pg/mL, P < 0.001; Figure 2B), and bacterial infection at admission (1.53 ± 0.63 pg/mL *vs.* 2.12 ± 0.79 pg/mL; P = 0.001).

The fT4 levels were positively correlated with TSH, age, INR, PCR, total bilirubin, and MELD score. A negative correlation was observed between fT4 levels and fT3, fT3/fT4 ratio, sodium and albumin. Significantly higher levels of fT4 were observed in Child-Pugh C patients as compared to Child-Pugh A (1.23 ± 0.24 ng/dL *vs.* 1.03 ± 0.24 ng/dL; P = 0.008) and Child-Pugh B ones (1.23 ± 0.24 ng/dL *vs.* 1.05 ± 0.19 ng/dL; P < 0.001; Figure 1C). The presence of ACLF was also associated with higher fT4 as compared to acute decompensation without ACLF (1.24 ± 0.22 ng/dL *vs.* 1.08 ± 0.23 ng/dL; P = 0.001; Figure 2C). Significantly lower levels of fT4 were observed in upper gastrointestinal bleeding (1.02 ± 1.02 ng/dL *vs.* 1.24 ± 0.23 pg/mL; P < 0.001).

The fT3/fT4 ratio was positively correlated with sodium, albumin levels, and fT3. A negative correlation was observed between fT3/fT4 and fT4, lNR, total bilirubin, CRP, MELD, and creatinine. Significantly lower fT3/fT4 values were observed in Child-Pugh C patients when compared to Child-Pugh A (1.26 ± 0.55 *vs.* 2.97 ± 0.99; P < 0.001) and Child-Pugh B ones (1.26 ± 0.55 *vs.* 2.10 ± 0.79; P < 0.001). Child-Pugh B patients also presented lower fT3/fT4 ratio than Child-Pugh A ones (2.10 ± 0.79 *vs.* 2.97 ± 0.99; P = 0.003; Figure 1D). Lower fT3/fT4 ratio was also associated with ACLF (1.24 ± 0.58 *vs.* 2.07 ± 0.92, P < 0.001; Figure 2D) ascites (1.47 ± 0.67 *vs.* 2.23 ± 0.96; P < 0.001), hepatic encephalopathy (1.64 ± 0.83 *vs.* 2.17 ± 0.95; P = 0.002), and bacterial infection at admission (1.37 ± 0.67 *vs.* 2.02 ± 0.93; P < 0.001). Significantly higher fT3/fT4 was observed in patients with upper gastrointestinal bleeding (2.23 ± 0.88 *vs.* 1.42 ± 0.76; P < 0.001).

Thyroid hormones were evaluated according to the etiology of liver disease (viral *vs*. nonviral and alcoholic *vs*. nonalcoholic). As compared to nonviral etiology, cirrhosis caused by chronic hepatitis B or C was associated with slightly higher fT3 (2.16 ± 0.82 pg/mL *vs.* 1.85 ± 0.75 pg/mL; P = 0.032) and lower fT4 (1.07 ± 0.22 ng/dL *vs.* 1.17 ± 0.23 ng/dL; P = 0.008), with no impact on TSH (0.98 μIU/mL *vs.* 1.42 μIU/mL; P = 0.152) and fT3/fT4 ratio (2.15 ± 1.02 *vs.* 1.66 ± 0.77; P = 0.096). On the other hand, when compared to nonalcoholic cirrhosis, cirrhosis of alcoholic etiology was associated with lower fT3 (1.78 ± 0.70 pg/mL *vs.* 2.10 ± 0.82 pg/mL; P = 0.025), but no significant differences regarding TSH were found (1.42 μIU/mL vs. ١.13 μIU/mL; P = 0.603), fT4 (1.15 ± 0.24 ng/dL *vs.* 1.10 ± 0.23 ng/dL; P = 0.353), and fT3/fT4 ratio (1.62 ± 0.71 *vs.* 2.02 ± 0.99, P = 0.282).

### Thyroid hormones and short-term prognosis in patients hospitalized for AD of cirrhosis

The overall 90-day mortality rate was 26.9%. Only two patients underwent liver transplantation within 90 days from admission. In the bivariate analysis, 90-day mortality was associated with older age (58.19 ± 12.57 *vs.* 51.83 ± 11.58; P = 0.011), ascites (84.4% *vs.* 32.2%; P < 0.001), hepatic encephalopathy (71.9% *vs.* 49.4%; P = 0.029), Child-Pugh C (75.0% *vs.* 25.3%; P < 0.001), ACLF at admission (59.4% *vs.* 10.3%; P < 0.001) and higher MELD score (22.12 ± 6.32 *vs.* 14.07 ± 4.92; P < 0.001). 90-day mortality was also related to higher creatinine (1.65 mg/dL *vs.* 1.00 mg/dL; P < 0.001), INR (1.54 *vs.* 1.38; P = 0.028), CRP (24.10 mg/L *vs.* 7.9 mg/L; P = 0.003), total bilirubin (3.70 mg/dL *vs.* 1.35 mg/dL; P < 0.001) and lower mean albumin (2.03 ± 0.53 g/dL *vs.* 2.47 ± 0.63 g/dL; P < 0.001) and sodium levels (132.69 ± 7.58 mEq/L *vs.* 136.59 ± 5.21 mEq/L; P = 0.010). Regarding thyroid hormones, patients who died exhibited higher median TSH (2.63 μIU *vs.* 1.10 μIU/mL; P = 0.009) and mean fT4 levels (1.26 ± 0.22 *vs.* 1.07 ± 0.22; P < 0.001), and lower median fT3 levels (1.44 pg/mL *vs.* 2.19 mcg/mL; P < 0.001) and mean fT3/fT4 ratio (1.17 ± 0.45 *vs.* 2.14 ± 0.91; P < 0.001), (Table 3).

**Table 3:** Factors associated with 90-day mortality among patients hospitalized for acute decompensation of cirrhosis

	Survivors (n = 87)	Deaths (n = 32)	P
Age (years), mean ± SD	51.83 ± 11.58	58.19 ± 12.57	0.011
Male gender, n (%)	63 (72.4)	22 (68.8)	0.695
Etiology of cirrhosis, n (%)			
Alcohol	30 (34.5)	13 (40.6)	0.536
Hepatitis C	39 (44.8)	09 (28.1)	0.100
Hepatitis B	3 (3.4)	2 (6.3)	0.610
Cryptogenic	5 (5.7)	3 (9.4)	0.442
Other	10 (11.5)	5 (15.6)	0.544
Previous decompensation, n (%)	52 (59.8)	21 (65.6)	0.561
Active alcoholism, n (%)	30 (34.5)	12 (37.5)	0.760
Complication at evaluation, n (%)			
Ascites	28 (32.2)	27 (84.4)	< 0.001
Hepatic encephalopathy	43 (49.4)	23 (71.9)	0.029
Gastrointestinal bleeding	60 (69.0)	7 (21.9)	<0.001
Bacterial infection	16 (18.4)	11 (34.4)	0.065
ACLF, n (%)	9 (10.3)	19 (59.4)	< 0.001
Laboratory data			
Leucocyte count (x 109), median (IQR)	7.14 (3.89-10.15)	7.57 (5.53-11.17)	0.290
Sodium (meq/L), mean ± SD	136.59 ± 5.21	132.69 ± 7.58	0.010
Creatinine (mg/dl), median (IQR)	1.00 (0.80-1.20)	1.65 (1.13-2.55)	< 0.001
INR, median (IQR)	1.38 (1.27-1.55)	1.54 (1.32-1.81)	0.028
Albumin (g/dL), mean ± SD	2.47 ± 0.63	2.03 ± 0.53	0.001
CRP (mg/L), median (IQR)	7.9 (3.70-28.40)	24.1 (5.75-108.25)	0.003
Total bilirubin (mg/dL), median (IQR)	1.35 (0.90-3.20)	3.7 (1.48-6.98)	< 0.001
Child-Pugh C, n (%)	22 (25.3)	24 (75.0)	< 0.001
MELD score, mean ± SD	14.07 ± 4.92	22.12 ± 6.32	< 0.001
Thyroid hormones			
TSH (μIU/mL), median (IQR)	1.10 (0.53-2.11)	2.63 (0.98-3.88)	0.009
fT3 (pg/mL), mean ± SD	2.19 ± 0.78	1.44 ± 0.52	< 0.001
fT4 (ng/dL), mean ± SD	1.07 ± 0.22	1.26 ± 0.22	< 0.001
fT3/fT4 ratio, mean ± SD	2.14 ± 0.91	1.17 ± 0.45	<0.001

SD: standard deviation; ACFL: acute-on-chronic liver failure; IQR: interquartile range; INR: international normalized ratio; CRP: C-reactive protein; MELD: Model for End-stage Liver Disease; TSH: thyroid stimulating hormone; fT3: free triiodothyronine; fT4: free thyroxine.

A stepwise forward logistic regression analysis was performed including the following variables with P ≤ 0.010 in the bivariate analysis: Child-Pugh C, MELD, ACLF, sodium, CRP, TSH, and fT3/fT4 ratio. Other variables already included in the prognostic models (ascites, hepatic encephalopathy, creatinine, total bilirubin, albumin, fT3, and fT4) were not included to avoid multicollinearity. In this analysis, 90-day mortality was independently associated with the MELD score (OR 1.166, 95% CI 1.054 – 1.290; P = 0.003), fT3/fT4 ratio (OR 0.242, 95% CI 0.080 – 0.734; P = 0.012) and TSH (OR 1.337, 95% CI 1.045 – 1.710; P = 0.021).

The regression formula for prediction of 90-day mortality was as follows:


$$\begin{array}{cc} & \text{ Predictive model }=0.157(\text{ MELD score })-1.428\\ & (fT3/fT4\text{ ratio })+0.292(TSH[\mu IU/mL])-1.154 \end{array}$$

The AUROCs of the predictive model, MELD score, fT3/fT4 ratio, and TSH for predicting 90-day mortality were 0.899 ± 0.031, 0.847 ± 0.041, 0.841 ± 0.039, and 0.658 ± 0.062, respectively.

**Figure 3:**
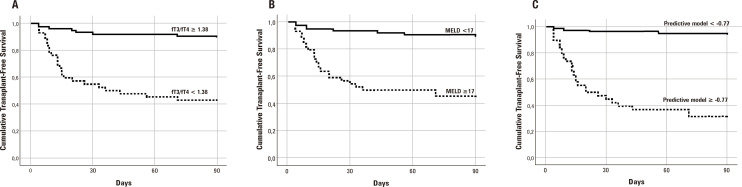
Cumulative 90-day transplant-free survival of hospitalized patients with cirrhosis according to MELD score and fT3/fT4. Kaplan-Meier survival probability was 89.5% in patients with fT3/fT4 ≥ 1.38 and 42.9% for subjects with fT3/fT4 < 1.38 (P < 0.001) (Figure 3A). The 90-day survival was 45.5% in patients with MELD ≥ 17 and 89.3% for subjects with MELD < 17 (Figure 3B). Figure 3C exhibits the Kaplan-Meier curves according to the best cutoff of the prognostic model devised from regression analysis. The 90-day Kaplan-Meier survival probability was 31.6% in patients with values of the predictive model ≥ -0.77 and 93.5% for patients with values < -0.77 (P < 0.001, long-rank test).

The best cutoffs for predicting 90-day mortality were chosen based on ROC curves. The Kaplan-Meier survival probability at 90 days was 89.5% in patients with fT3/fT4 ≥ 1.38 and 42.9% for subjects with fT3/fT4 < 1.38 (P < 0.001), (Figure 3A). The best MELD score cutoff to predict 90-day mortality was 17. The Kaplan-Meier survival probability at 90 days was 45.5% in patients with MELD ≥ 17 and 89.3% for subjects with MELD <17 (Figure 3B). Regarding the predictive model, the best cutoff was -0.77. The 90-day Kaplan-Meier survival probability was 31.6% in patients with values of the predictive model ≥ -0.77 and 93.5% for patients with values < -0.77 (Figure 3C).

For the prediction of 90-day mortality, fT3/fT4 at a cutoff of 1.38 showed a sensitivity of 75% and a specificity of 79%, a negative predictive value of 90%, and a positive predictive value of 57%. MELD score at a cutoff of 17 showed a sensitivity of 75% and a specificity of 77%, a negative predictive value of 89%, and a positive predictive value of 55%. Better results were obtained by using the devised predictive model at a cutoff of -0.77, with a sensitivity of 84% and a specificity of 86%, a negative predictive value of 94%, and a positive predictive value of 68%.

## DISCUSSION

The natural course of liver cirrhosis is often punctuated by life-threatening complications requiring hospitalization. Although the clinical phenotype of these acute decompensation episodes varies significantly, the systemic consequences tend to be similar across individuals, usually reflecting the severity of liver dysfunction or the acute insult that precipitated clinical worsening (23). Studying these systemic abnormalities in patients with complicated cirrhosis may be helpful in prognostication as more specific models have presented several limitations (24,25).

In the present study, abnormal TSH levels were observed in 28% of the sample patients. In addition, TSH was positively correlated with CRP, and higher TSH levels were observed in Child-Pugh C patients. Although previous studies reported conflicting results regarding the impact of liver disease on TSH levels, the majority of data indicates higher TSH levels in patients with cirrhosis as compared to controls (8,11,26). Furthermore, TSH appears to increase parallel with the severity of liver dysfunction (26,27).

FT3 was lower than the reference value in 40% of the patients and correlated with several variables related to the severity of liver disease. In addition, lower fT3 was also noted in patients with severe complications such as bacterial infections, hepatic encephalopathy, and ACLF. These findings are in agreement with several previous studies that demonstrated that low T3 or fT3 patterns is the most common thyroid abnormality in liver cirrhosis (6,13,27-30). In patients with outpatient stable cirrhosis, this pattern is likely related to impaired hepatic conversion of T4 to T3 as a result of reduced deiodinase 1 activity (31). However, in acute decompensation of cirrhosis, a more typical EES is expected and multiple mechanisms are involved in its pathogenesis, including alterations in the iodothyronine deiodinases, thyroid-stimulating hormone secretion, thyroid hormone binding to plasma protein, transport of thyroid hormone in peripheral tissues, and thyroid hormone receptor activity 9.

In this study, fT4 levels were in the normal range in 96% of the patients. However, contrary to fT3, fT4 levels were significantly higher in patients with evidence of more advanced liver disease and in those with ACLF. The influence of liver disease on fT4 levels is less clear. Burra et al. showed that more than half of patients with cirrhosis had fT4 concentration above the normal range, but no correlation was observed between fT4 and any of the liver function tests examined (32). This low-fT3-high-fT4 pattern was also observed in other studies and, at least in part, resulted from reduced T4 to T3 peripheral conversion (8,31). Interestingly, in EES, total T4 levels are normal or even increased in early stages, but tend to fall as the disease progresses (9). However, fT4 appears to be less affected and may be normal in cases of EES (9). We believe that the association of higher fT4 with the more severe disease observed here may reflect the dynamic of this hormone described in EES, as our patients were evaluated very early in the course of the disease. In addition, acute complications with concomitant EES also can influence thyroid hormone levels and it having a significant impact on the etiology of liver cirrhosis, although unlikely, cannot be ruled out.

A lower fT3/fT4 ratio was also related to markers of the intensity of liver dysfunction and the severity of a precipitant event of acute decompensation episode. The fT3/fT4 ratio is considered an indicator of peripheral deiodinase activity (33) and the lower fT3/fT4 ratio was recently related to EES in the pediatric population (10). It appears to be especially interesting in acute decompensation of cirrhosis as our data showed that lower fT3 and higher fT4 were associated with the severity of liver disease. A recent study including 272 patients hospitalized for complications of cirrhosis showed that a fT3/fT4 ratio correlated with variables of severity of liver disease such as MELD, CLIF-OF, and Child-Pugh, and was significantly lower in patients with AD as compared to stable outpatients, and in ACLF as compared to AD 13. However, although fT3 was an independent predictor of prognosis, fT3/fT4 ratio, fT4 levels, and TSH were not included in this survival analysis.

In the present study, higher TSH and lower fT3/fT4 ratio, along with MELD score, were independently associated with 90-day mortality. In addition, higher fT4 was associated with mortality in the bivariate analysis. As mentioned above, higher TSH was related to more advanced liver disease (26,27). However, contrary to the expected, published data so far found no association between higher TSH and mortality in liver cirrhosis (11,27,29,34). This could be explained at least in part by the dynamics of thyroid hormones in EES. In the first hours of a severe illness, T3 levels decrease but TSH levels are normal or even elevated (35). With the progression of the disease, TSH levels decline as a result of a decrease in TRH release from the hypothalamus (35). In the present study, all patients were evaluated early upon hospitalization, therefore less impact of the acute complication on TSH levels is expected. Previous reports indicated that low T3 or fT3 were related to mortality in patients with cirrhosis (13,29,34-38). There is little data regarding fT4 and survival regarding cirrhosis. Contrary to our findings, Tas et al. showed that lower fT4 was associated with mortality in intensive care patients with cirrhosis (29). This discrepancy may be justified by the profile of patients evaluated in both studies. Here, we included patients when admitted to the emergency room and the Turkish study evaluated only patients admitted to the intensive care unit, possibly with more severe complications and in the latter stages of EES (29). This is the first study investigating the fT3/fT4 ratio as a prognostic marker in cirrhosis. The combination of fT3 and fT4 in one index was able to maximize the prognostic impact of the individual variables. In this study, a new prognostic model was devised based on the regression analysis, including the three independent prognostic factors: MELD score, fT3/fT4 ratio, and TSH. This new model showed higher AUROC and significantly better discriminative capabilities than each of the variables alone, allowing the early identification of a group of patients with a very poor prognosis and another with a low probability of death within 90 days.

We acknowledge some limitations to our analysis. The relatively small number of patients evaluated could limit our ability to generalize these findings to other populations. It is important to validate our findings before incorporating them into clinical practice. Another limitation that we should highlight is the fact that we included a very heterogeneous population in distinct clinical scenarios. Thyroid hormone changes in acute complications of chronic diseases are complex and dynamic; thus, test results should be interpreted with caution in individual cases. As we studied patients early in the course of the acute decompensation of cirrhosis, our findings might not be extrapolated to other clinical scenarios such as intensive care units and liver transplantation.

In conclusion, in patients recently admitted for AD of cirrhosis, higher TSH and fT4 and lower fT3 and fT3/fT4 ratios were associated with variables related to the severity of liver dysfunction. TSH levels and fT3/fT4 ratio were independently associated with short-term prognosis. A new prognostic model including MELD, TSH, and fT3/fT4 ratio achieved high accuracy in predicting short-term mortality and might be of clinical value as a prognostic tool.
